# Supplementary material for: Multimodal imaging and functional analysis of the chick NMDA retinal damage model
Source: PLoS One. 2021 Sep 7;16(9):e0257148. doi: 10.1371/journal.pone.0257148 (PMC8423281; doi:10.1371/journal.pone.0257148)
Supplement: S1 File — (ZIP) [file pone.0257148.s004.zip › SD-OCT/C200727/011/D15-PWk3/C200727-011_OS_V_14X14_0_REGAVG0000053/Manual_PreDefineFiveByFiveGridRectangularRetina_1.pdf]

Five By Five Grid Template Report

**Patient ID**	C200727	**Name**	See InVivoVue
**Exam Date**	See InVivoVue	**Exam Time**	See InVivoVue
**Eye**	OS	**Averaged**	N/A
**Depth (mm)**	1.65	**Depth Samples**	1024
**Pitch (um/pix)**	35.000 x 35.000 x 1.608	**Refractive Index**	See InVivoVue
**Scan Type**	Rectangular 14.000mm x 14.000mm x 1.647mm , 400 x 400 x 1024		

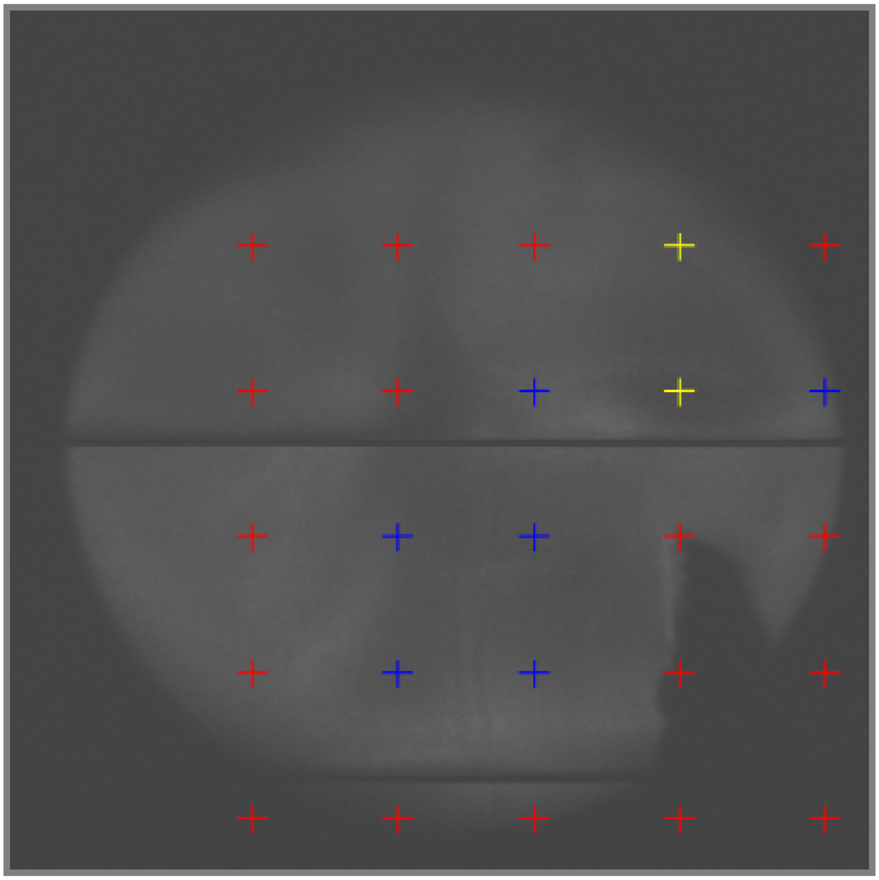

Thickness Metrics

Analysis	Average(mm)	Minimum(mm)	Maximum(mm)	STDEV(mm)
**IRNFL - RPE**	0.2736	0.2577	0.2833	0.0098
**IRNFL - ORNFL**	0.0408	0.0187	0.0668	0.0174
**ORNFL - OPL**	0.1569	0.1336	0.1791	0.0186
**OPL - ETPRS**	0.0563	0.0530	0.0605	0.0029
